# ACTIVITIES OF DAILY LIVING RECOVERY IN HOME HEALTH PATIENTS WITH DIABETES

**DOI:** 10.1093/geroni/igad104.3761

**Published:** 2023-12-21

**Authors:** Katelyn Webster-Dekker, Yvonne Lu, Susan Perkins, Jennifer Ellis, Laurie Otis, Rebecca Winton, Eileen Hacker

**Affiliations:** Indiana University School of Nursing, Indianapolis, Indiana, United States; Indiana University School of Nursing, Indianapolis, Indiana, United States; Indiana University School of Medicine, Indianapolis, Indiana, United States; Aveanna Healthcare, Atlanta, Georgia, United States; Aveanna Healthcare, Atlanta, Georgia, United States; CenterWell Home Health, Atlanta, Georgia, United States; The University of Texas MD Anderson Cancer Center, Houston, Texas, United States

## Abstract

Older adults with diabetes are at high risk for impairments in their ability to perform activities of daily living (ADLs). Home health (HH) services help patients regain their ability to perform ADLs after being hospitalized, but there may be disparities in degree of ADL improvement based on characteristics such as race/ethnicity. We aimed to identify factors associated with improvements in ADLs from the start of HH care to discharge in older adult (age ≥65) patients with diabetes receiving HH. This secondary analysis used Outcome and Assessment Information Set-D data collected between October 1, 2021, and March 31, 2022 in the Southern U.S by a HH agency. We used multiple linear regression to examine factors associated with improvement in ADL performance. The sample (n=1350) was 55% female and 76% White, with a mean age of 76.3 (SD 7.3). Ninety-seven percent of patients improved their ADL score from start of HH care to discharge. Black/African American race (b= -0.33) and having bowel incontinence or an ostomy (b= -0.51) were associated with less ADL improvement. Having a caregiver who needed training/support (b= 0.44) or was unlikely to provide assistance (b= 0.78), the presence of a surgical wound (b= 0.52), pain that interfered with activity (b= 0.46), confusion (b= 0.30), and better scores in prior functioning (b= 0.13) at the start of HH were associated with greater improvement in ADLs upon discharge from HH. These findings require further investigation, but indicate Black patients experienced disparities in ADL improvement which should be addressed.

